# Attitudes and Expectations of Health Care Professionals Toward App-Based Therapy in Patients with Osteoarthritis of the Hip or Knee: Questionnaire Study

**DOI:** 10.2196/21704

**Published:** 2020-10-28

**Authors:** Johanna Theresia Biebl, Stephan Huber, Marzena Rykala, Eduard Kraft, Andreas Lorenz

**Affiliations:** 1 Department of Orthopaedics, Physical Medicine and Rehabilitation University Hospital, LMU Munich Munich Germany; 2 Kaia Health GmbH Munich Germany

**Keywords:** mobile health, digital health, self-management, osteoarthritis, smartphone, patient education, exercise therapy

## Abstract

**Background:**

The use of mobile health (mHealth) apps is becoming increasingly widespread. However, little is known about the attitudes, expectations, and basic acceptance of health care professionals toward such treatment options. As physical activity and behavior modification are crucial in osteoarthritis management, app-based therapy could be particularly useful for the self-management of this condition.

**Objective:**

The objective of the study was to determine the expectations and attitudes of medical professionals toward app-based therapy for osteoarthritis of the hip or knee.

**Methods:**

Health care professionals attending a rehabilitation congress and employees of a university hospital were asked to fill out a questionnaire consisting of 16 items. A total of 240 questionnaires were distributed.

**Results:**

A total of 127 participants completed the questionnaire. At 95.3% (121/127), the approval rate for app-based therapy for patients with osteoarthritis of the hip or knee was very high. Regarding possible concerns, aspects related to data protection and privacy were primarily mentioned (41/127, 32.3%). Regarding potential content, educational units, physiotherapeutic exercise modules, and practices based on motivation psychology were all met with broad approval.

**Conclusions:**

The study showed a high acceptance of app-based therapy for osteoarthritis, indicating a huge potential of this form of treatment to be applied, prescribed, and recommended by medical professionals. It was widely accepted that the content should reflect a multimodal therapy approach.

## Introduction

According to the World Health Organization, mobile health (mHealth) refers to medical procedures in private and public health care that are applied by means of mobile devices using various technologies [1]. Mobile devices such as smartphones are widely used in the population and have a large number of sensors that can measure vital signs and other health-related data and display patients’ progress [2]. Combining sensor data with actively provided information by users and interaction with health care professionals (HCPs) opens up new possibilities for diagnosis and intervention [3].

Osteoarthritis (OA) is the most common joint disease and can lead to severe pain, impaired physical activity, and severely restricted health-related quality of life [4]. The incidence and prevalence of OA will continue to rise in the future due to an aging society [5]. The importance of patient education and physical exercise as part of therapy is undisputed [6]. In their systematic review, Hagen et al [7] showed that in the field of community-based care, only 38.8% of patients received the recommendation to perform physical exercise or a corresponding prescription. Patient education and advice on self-management strategies were offered to only 35.4% of the patients. Thus, there is a discrepancy between accepted recommendations and the reality of care for patients with OA.

Educational content and suitable exercises could also be taught using an app. It has been shown that health apps are well suited for implementing sustainable behavior changes in the daily life of chronically ill patients [8]. App-based therapeutic options are already available for various chronic conditions, including insomnia, diabetes, chronic knee pain, and low back pain [9-13]. Many of these apps contain communication possibilities and offer sources of information and options for documentation, such as diaries. In addition, planning tools such as appointment reminders and medication schedules are intended to improve adherence to therapy. Aspects that also play a role in the management of OA of the hip and especially of the knee joint, such as weight reduction, have already been successfully addressed in other contexts using app-based approaches [14,15]. Regarding telemedical care for OA, a Swedish research group has translated a conventional OA self-management program—the Management of Patients with Osteoarthritis (BOA) program—into a digital form called Joint Academy. The BOA program was developed on the basis of existing evidence, national and international treatment guidelines, and patient interviews [16]. In the digital form, there is a platform for patients that offers exercises, physiotherapeutic counselling, support from other affected people, and educational content [17]. Participants who used the Joint Academy platform approximately 5 days a week showed an improvement in pain and physical functioning [18]. Another existing digital therapy is the so-called Hinge Health program designed for patients with chronic knee pain, including patients with OA. Among other components, this program offers active exercises in which patients wear portable bands with motion sensors, allowing feedback on their exercise performance [11,19]. In their randomized controlled trial, Mecklenburg et al [11] detected that patients with chronic knee pain who were treated with the Hinge Health program for 12 weeks had significantly better results in terms of pain, physical functioning, surgery, and understanding of the disease than a control group.

Because app-based therapy is a novel technique, interest in the acceptance and expectations of medical professionals regarding app-based therapy is growing. For instance, an Australian study [20] found that approximately two-thirds of the participating general practitioners used apps themselves within their professional activities, and approximately one-half of the respondents recommended the use of apps to their patients. In another study [21], interviews with primary care physicians identified barriers and facilitators for the implementation of apps for the self-management of diabetes. Moreover, there has been research regarding the attitudes of HCPs toward app-based therapy for depression. While only 21.1% had used app-based therapy with their patients before, 66.0% believed that outcomes would improve if apps were integrated into the treatment of depression [22]. Kessel et al [23] conducted an online survey specifically to evaluate the attitudes and expectations of HCPs regarding the use of telemedicine and apps in the field of oncology, and they detected a broad overall support for these forms of care: 88.9% of respondents considered telemedicine to be useful and 84.3% were in favor of an oncological app in addition to standard care. However, to our knowledge, expectations of HCPs regarding app-based therapy for patients with OA have not yet been recorded in a structured way. In the field of musculoskeletal diseases, a recent review by Najm et al [24] showed that the involvement of physicians and other medical professionals in the development and design of apps has been low to date and that their increased participation would be preferable.

Under these circumstances, this study aimed to determine the expectations and attitudes of medical professionals toward app-based therapy for OA of the hip or knee joint. Based on the results, an app is to be developed that meets the expectations of potential mediators of the app and takes into account their clinical experience.

## Methods

The study was approved by the Ethics Committee of Ludwig Maximilian University of Munich (LMU Munich), Munich, Germany (reference number 19-627). Written informed consent was obtained from all participants before the survey began.

### Study Design

A questionnaire with 16 main items was developed based on recommendations by Langbecker et al [25]. After literature research, we conducted interviews with health experts from different professions and specialties. The collected information was categorized and structured. Based on these data, national guidelines [26,27], and the care standards of our university hospital (LMU Munich), the questionnaire’s content was defined by an interdisciplinary team of physicians from various disciplines, psychologists, physiotherapists, and persons knowledgeable in the development of medical mobile apps. The questionnaire was then pretested on a collective of HCPs with regard to comprehensibility and clarity.

Of the 16 questions, 11 were closed and 5 were semiopen. Multiple answers were possible. Two of the main items consisted of 12 and 5 subitems, respectively, each of which was to be assessed on a 5-point Likert scale (1=not useful, 2=rather not useful, 3=partially useful, 4=rather useful, 5=useful). One of the items was used to collect personal data and consisted of four subitems. For some questions, it was possible to specify “no comment” as the answer.

The questions related to possible advantages and disadvantages of the app-based therapy, possible educational content, meaningful exercises, and possible problem areas, as well as to the idea of embedding the app in existing technical systems and possible connections (eg, to so-called wearables—devices that can be worn on the body and use computer technologies).

The participants were asked to indicate their gender, length of professional experience, occupation, and field of activity. Professional experience was categorized in 5-year steps (less than 5 years, 5-10 years, 10-15 years, 15-20 years, 20-25 years, 25-30 years, and more than 30 years).

The questionnaire survey was conducted in an anonymous form. The questionnaires (see Multimedia Appendix 1) were handed out to employees at the University Hospital, LMU Munich, and to attendees at a rehabilitation congress at the same institution. Inclusion criteria were having a self-reported degree in a regulated medical profession and age over 18 years. A total of 240 questionnaires were handed out. No incentives were offered for participation.

In order to avoid a selection bias toward individuals with higher technological affinity, the survey was deliberately distributed as a paper questionnaire. Nevertheless, we have based the presentation of the results, as far as possible, on the Checklist for Reporting Results of Internet E-Surveys (CHERRIES) [28] to comply as closely as possible with standards in this field of research.

### Statistical Analysis

The data were analyzed using descriptive statistics. If no information was provided for a question, it was taken into account when calculating the percentages and is indicated accordingly. The number of participants who had answered the respective question is shown in parentheses.

In order to detect possible correlations between years of professional experience and approval of app-based therapy, several contingency tables were analyzed. First, the levels of work experience were dichotomized with 6 different cutoffs of years of professional experience, and 6 2x2 contingency tables were built. Second, the level of work experience was dichotomized as individual groups of professional experience versus all other experience levels, and 6 more 2x2 tables were created. All contingency tables were analyzed using the Fisher exact test. Statistical analysis was conducted using SPSS software (version 21.0; IBM Corp).

## Results

A total of 127 HCPs submitted completed questionnaires (response rate 52.9%). The characteristics of the sample are listed in Table 1.

**Figure 1:**
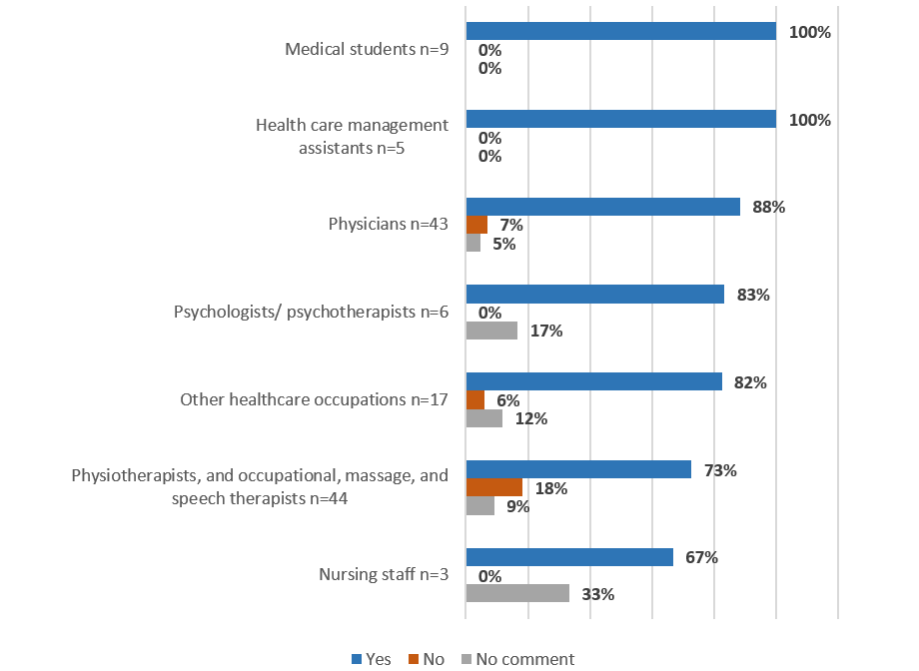
General approval of app-based therapy for osteoarthritis categorized according to profession.

In response to the question of whether they would generally recommend an app-based therapy to their patients for the treatment of OA of the knee or hip, 89.0% (105/118) replied yes. Figure 1 provides an illustration of the answer to this question divided according to patients’ occupational groups. There was no statistically significant difference in participants’ attitude toward recommendation of an app-based therapy based on their years of professional experience (Table 2).

**Table 1:** Sample characteristics (N=127).

Characteristics			n (%)	
**Gender**				
	Female			78 (61.4)
	Male			49 (38.6)
**Professional experience (years)**				
	Less than 5			36 (28.3)
	5-10			21 (16.5)
	10-15			17 (13.4)
	15-20			12 (9.4)
	20-25			13 (10.2)
	25-30			12 (9.4)
	More than 30			16 (12.6)
**Occupation/training**				
	Physicians			43 (33.9)
		Hospital sector		24 (18.9)
		Outpatient sector		13 (10.2)
		Medical activity in other areas		6 (4.7)
	Nursing staff			3 (2.4)
	Therapeutic occupations			44 (34.6)
		Occupational therapists		6 (4.7)
		Massage therapists		3 (2.4)
		Physiotherapists		33 (26.0)
		Speech therapists		2 (1.6)
	Health care management assistants			5 (3.9)
	Psychologists/psychotherapists			6 (4.7)
	Medical students			9 (7.1)
	Other health care professions			17 (13.3)
**Field of activity**				
	Surgical medicine			11 (8.7)
	Conservative medicine			84 (66.2)
	Both conservative and surgical medicine			14 (11.0)
	No specification provided			18 (14.2)

**Table 2:** Differences in approval of app-based therapy according to level of professional experience. *P* values are provided for comparison of each group to all other groups of professional experience.

Levels of professional experience (years)	*P* value^a^
<5	.51
5-10	>.99
10-15	>.99
15-20	>.99
20-25	.13
25-30	>.99
>30	.38
<10	.39
<15	.24
<20	.39
<25	.49

^a^Calculated using two-sided Fisher exact test.

The specification that an app should be used in addition to conventional therapy was accepted by 95.3% (121/127).

In regard to the arguments against the use of an app to support the treatment of OA, 32.3% (41/127) of the participants cited data protection and data privacy problems as their main concerns. Concerns about the safety of patients were cited by 24.4% (31/127) of participants, and lack of evidence was cited by 20.5% (26/127). While 14.2% (18/127) of participants feared that the use of an app might impair the doctor-patient relationship, 30.7% (39/127) of participants stated that they had no reservations.

**Figure 2:**
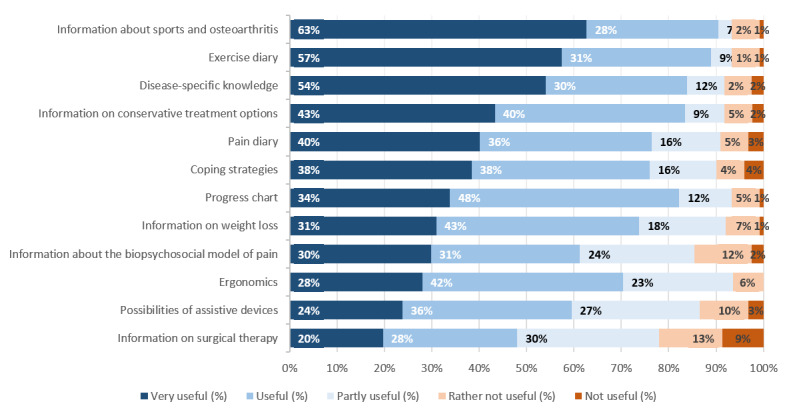
Health care professionals´ opinions regarding possible educational content for an osteoarthritis app.

Concerning perceived advantages, 67.7% (86/127) of participants saw the flexible access to the information source as an advantage of app-based OA therapy. The flexible use of exercises was viewed as an advantage by 78.7% (100/127), while 63.0% (80/127) perceived the strengthening of competence in disease management to be a positive aspect of app-based therapy. The independence of appointments with health care providers was seen as an opportunity by 44.9% (57/127) of participants. It was stated by 25.2% (32/127) of participants that they considered a reduction in the number of prescriptions that might result from providing patients with an app to be valuable. The health education content that was emphasized by health care professionals as being potentially important content for an OA app is shown in Figure 2.

**Figure 3:**
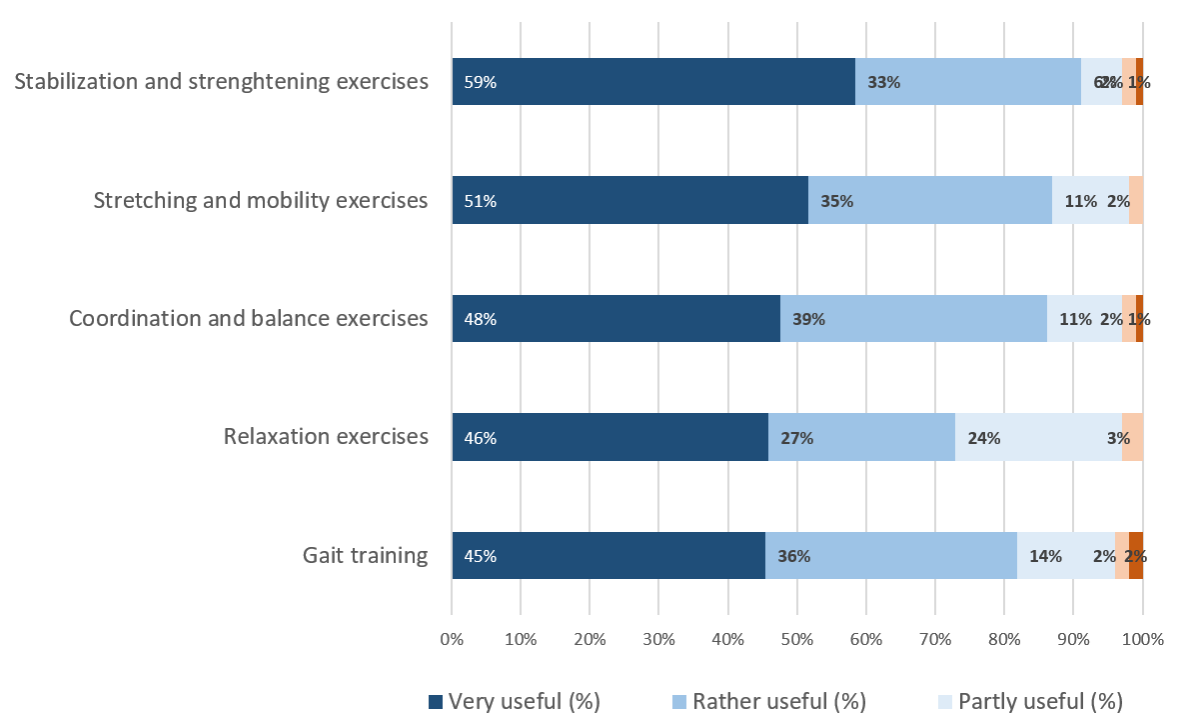
Health care professionals’ opinions regarding different types of exercises.

Participants’ attitudes toward possible exercise modules are shown in Figure 3. Approximately 77.2% (98/115) of the participants said they would welcome the integration of coaching procedures into the app, but 14.8% (17/115) said they were against it. It was believed by 76.0% (77/115) of participants that patients should receive feedback (eg, via SMS text messaging), while 33.0% (38/115) did not recommend this function.

**Figure 4:**
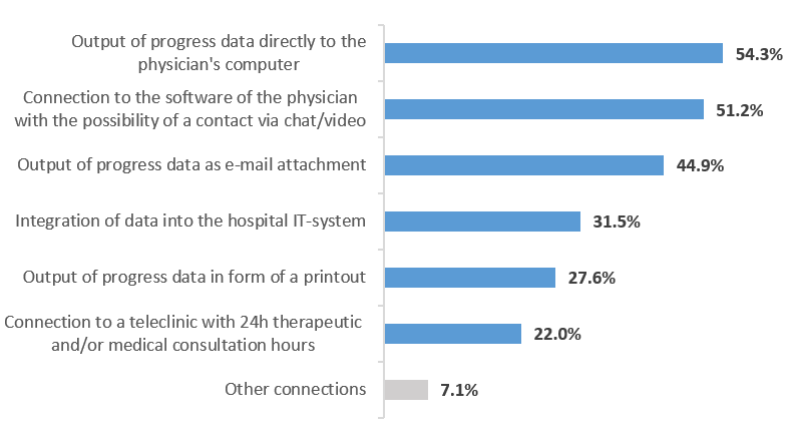
Consent to telemedical connections. IT Information Technology; 24h: 24-hour.

Furthermore, participants were asked about their opinion on the extent to which an OA app should be connected to other telemedical systems. The results of this question are shown in Figure 4.

In addition, the participants were asked for which patient groups an OA app could be particularly useful. Here, 31.5% (40/127) stated that a corresponding app could be of preventive value for all persons with a BMI above 25 kg/m^2^ and an age over 65 years. Free access for all individuals who want to remain active in old age was advocated by 54.3% (69/127). Approximately 50.4% (64/127) of participants were in favor of the use of an app for patients with OA after prior consultation with a general practitioner, orthopedist, physician, or pain therapist. Among the participants, 22.8% (29/127) were in favor of using an app for all OA patients who had previously received at least 18 therapy sessions with an outpatient physiotherapist, while 48.8% (62/127) believed that patients should be treated with an OA app after 18 physiotherapy units with instruction of the exercises included in the app. The approval rate for patients with OA who have already received multimodal therapy to use an app for continuous follow-up and therapy was 44.1% (56/127).

Of the devices that could potentially improve the app’s offering, 70.9% (90/127) of participants named fitness wristbands. The connection to a digital scale (eg, to check weight reduction) was considered useful by 37.0% (47/127) of participants. A connection to a blood pressure monitor for measurement at home was supported by 37.8% (48/127) of participants, while 25.2% (32/127) found a connection to a blood glucose meter useful. Approximately 15.0% (19/127) of participants stated that they thought that connecting external devices would not enrich the app.

## Discussion

### Principal Findings

Our survey showed that the vast majority of the HCPs surveyed, regardless of their prior professional experience or their discipline, were in favor of using a medical mobile app for patients with OA of the hip or knee. This suggests that HCPs would be likely to integrate well-developed medical apps into therapeutic regimens for patients with OA. The findings also indicate the large, untapped potential of HCPs to raise awareness of mHealth apps and to guide such novel treatment approaches.

We had expected that practitioners with a longer history of treating patients “conventionally” would be more cautious about this novel form of therapy. This assumption was also based on previous research that showed a rather reserved attitude of older HCPs regarding the use of mobile apps in their everyday work [29]. In our study, however, the different groups of professional experience did not differ significantly in their approval of app-based therapy.

Peeters et al [30] stated that patients who use technology to cope with their complaints have more disease-specific knowledge and a better understanding of their condition. However, it is precisely those patient groups who rarely use these resources who would benefit most from app-based information services and interventions [31]. In a survey by Rasche et al [32], users of health apps and general apps who were over 60 years old indicated that they obtain information about apps from family and friends, the internet, digital distribution platforms (eg, the App Store), magazines, television, and experts, with experts being the least used source of information. Therefore, medical professionals could play an important mediating role by reducing the inhibitions of chronically ill patients to use apps to manage their condition with a medical app.

Regarding the arguments against the use of digital care for patients with OA, data privacy issues were most frequently cited. These concerns should be taken seriously, and respective concepts for data privacy protection should be applied and presented in a transparent and comprehensible manner for both experts and users. The General Data Protection Regulation, which is now valid in the European Union, was developed to ensure transparency and reliability in the use of personal data. Even if there are still some uncertainties regarding the concrete practical implementation, there is a clear set of rules for the use of data with regard to app-based therapy [33]. Considerations for patient safety were also mentioned. Accordingly, there should be comprehensive concepts for patient protection. These could include 24-hour customer support, an integrated evaluation of red flags, and clear instructions on when to seek further medical help. Furthermore, vigilance systems can be implemented by manufacturers to collect available information on the safety profile of their apps in real-world use.

In addition to the interaction between physician and patient, Miyamoto et al [34] identified the integration of apps into existing health care services as a key element in initiating behavior changes in patients. Patients participating in the study wanted their collected data to be put into the context of their existing medical records using health apps so that they could receive individual and optimal medical advice based on the synopsis of their findings [34]. However, with regard to embedding an app in existing systems, a relatively large number of participants in our survey showed a certain reluctance. This could possibly be due to fears that app-based interactions with patients would be incalculable, difficult to plan, and involve additional work. In order to achieve wide acceptance, certain concepts might be advantageous to ensure that the individual practitioner is not confronted with unexpected, urgent requests with a direct need for action, even outside of office hours. Here, for example, a central primary contact who could process user requests could be established by the provider of the app.

The extent to which medical professionals advocate for the use of coaching strategies and individualized feedback (eg, via SMS text messaging) was another item in our questionnaire. More than two-thirds of the participants supported this. Behavior therapy strategies could help patients to learn how to initiate sustainable behavior changes on the one hand and how to deal with their illness on the other. For this purpose, an app design would be conceivable in which positive feedback, rewards for reaching previously defined goals, assistance with motivation problems, and the possibility of contacting experts or other interested parties could be integrated [35]. Frequently, therapeutic strategies developed for personal interaction between practitioner and patient are integrated into apps. To what extent modifications are useful and necessary here should be the subject of further research. In addition, the existing high drop-out rate in app-based therapy, as described by Krebs and Duncan [36], could possibly be overcome using approaches based on motivation psychology.

The active participation of medical professionals in the development of digital health apps would be helpful and desirable to ensure that HCPs’ expectations are met. Noergaard et al [37] underlined the great value of participatory development of health care services. In addition to the involvement of experts, the participation of patients in the development of digital services is essential [37]. Therefore, as a next step, surveys should be conducted with people affected by OA in order to gain precise knowledge of their needs and expectations. Findings from these future studies might serve to increase the acceptance and adherence among patients, which might potentially increase recommendations by HCPs when implemented.

Based on the findings of our survey, the following content should be considered when developing an app: (1) knowledge units, (2) exercise modules that cover a wide physiotherapeutic spectrum, and (3) psychological content in the form of motivation-promoting strategies and relaxation techniques. A combination of the various components of medical treatment, pain psychological strategies, physical activity, and patient information would represent a multimodal therapy approach and reflect current evidence and national guidelines [26,38-40]. Conceptual considerations of the biopsychosocial model of pain should also be included [41]. To our knowledge, there is currently no app for the indication of OA that completely covers all of these aspects and meets the declared expectations of HCPs.

For OA of the hip and knee joint, the 6-minute walking test is an important assessment tool. Stienen et al [42] evaluated an app-based 6-minute walking test for patients with degenerative diseases of the lumbar spine, which proved to be highly reliable with the results of the usual, nondigital execution of the test. In addition to other assessments, this test is also an important tool in the evaluation and follow-up of OA of the hip or knee joint [43,44]. The embedding of such assessment tools into an OA app could make a decisive contribution to the high quality of the app. Data show that for patients with OA of the knee, walking can have a beneficial effect on symptoms and functioning [45,46]. As a strategy to promote regular walking, tools just as pedometers could be integrated into an OA app.

### Limitations

One limitation of our study is the rather low participation of physicians who perform surgery in the survey population. This could explain why knowledge units about surgical options met with the least approval. Furthermore, the survey was conducted at a congress for rehabilitation medicine. This resulted in a certain preselection of participants in terms of the specialties of participants and a bias toward more academically oriented professionals choosing to attend an academic conference. Due to the survey having partially closed questions, a certain loss of information and a selection bias cannot be excluded. Other approaches, most notably qualitative methods, might lead to different outcomes when assessing the expectations of HCPs toward apps for OA. Furthermore, the high approval rate for app-based therapy may have been influenced by the fact that, as is known, people who are interested in a certain topic are more likely to participate in a corresponding survey [47].

### Conclusions

In our survey, there is a very positive attitude of HCPs toward app-based therapy for patients with OA of the hip or knee, indicating untapped potential in the development of an appropriate app. Because HCPs, in principle, see great opportunities in app-based therapy, well-thought-out, secure apps should stand a great chance of being recommended and used in practice. It turned out that an app structure with various modules consisting of knowledge transfer, physical exercises, and practices based on motivation psychology was widely supported. Future studies in the field should address patients’ expectations regarding mHealth treatments for OA to ensure these expectations are known and met.

## Appendix

Multimedia Appendix 1 Questionnaire (English version).

## Abbreviations

BOA: Management of Patients with Osteoarthritis program
HCP: health care professional
LMU Munich: Ludwig Maximilian University of Munich
mHealth: mobile health
OA: osteoarthritis
