# Supplementary material for: Attitudes and Expectations of Health Care Professionals Toward App-Based Therapy in Patients with Osteoarthritis of the Hip or Knee: Questionnaire Study
Source: JMIR Mhealth Uhealth. 2020 Oct 28;8(10):e21704. doi: 10.2196/21704 (PMC7657727; doi:10.2196/21704)
Supplement: Multimedia Appendix 1 [file mhealth_v8i10e21704_app1.doc]

Dear colleagues,

We plan to develop a healthcare app for patients with osteoarthritis of the hip and/or knee for smartphone and tablet. We would be interested to know whether you think such an app could be a useful addition to the therapy of osteoarthritis and what content you consider important.

We would be very pleased if you could fill out the following questionnaire (time required 5-10 minutes). Your data is, of course, anonymous and will be treated in strict confidence.

Thank you very much for your support!

1. **Do you treat patients with osteoarthritis?**

- Yes
- No

1. **Do you, in general, consider a smartphone app to support therapy for osteoarthritis patients, e.g. through educational measures, useful?**

- Yes
- No

1. **Would you recommend to your patients the use of an app for osteoarthritis in addition to conventional therapy?**

- Yes
- No

1. **What are, in your opinion, arguments against the use of an app as a supportive treatment for patients with osteoarthritis (multiple answers possible)?**

- No additional benefit
- Privacy issues/ data protection issues
- Legal concerns
- Impairment of the physician-patient relationship
- Concerns for patient safety
- Lack of evidence
- I have no reservations, in my opinion there are no arguments against the use of an app for these patients

1. **What other concerns, not mentioned above, can you think of?**

- None
- The following:

1. **What advantages of an osteoarthritis app do you see for the patient? (multiple answers possible)**

- None
- Strengthening the competence for disease management
- Flexible source of information
- Flexible use of exercises
- Less prescriptions for physiotherapy/ occupational therapy necessary
- Independence from appointments with health care providers like physiotherapists

1. **Which advantages not mentioned above can you think of?**

- None
- The following:

_____________________________________________________________________

1. **Which contents of an app for disease-specific knowledge modules do you find useful?**

|  | Useless | Rather useless | Partly useful | Rather useful | Very useful |
| --- | --- | --- | --- | --- | --- |
| Disease-specific knowledge |  |  |  |  |  |
| Coping strategies |  |  |  |  |  |
| Bio-psycho-social model of pain |  |  |  |  |  |
| Pain diary |  |  |  |  |  |
| Physical exercise diary |  |  |  |  |  |
| Information on weight loss |  |  |  |  |  |
| Graphic display of progress data |  |  |  |  |  |
| Ergonomics |  |  |  |  |  |
| Options for the supply of aids |  |  |  |  |  |
| Information on conservative therapy options |  |  |  |  |  |
| Information on surgical therapy options |  |  |  |  |  |
| Information on sports and osteoarthritis |  |  |  |  |  |

1. **Which exercises and contents do you consider useful?**

|  | Useless | Rather useless | Partly useful | Rather useful | Very useful |
| --- | --- | --- | --- | --- | --- |
| Stabilisation/strengthening exercises |  |  |  |  |  |
| Stretching/mobility exercises |  |  |  |  |  |
| Coordination/balance exercises |  |  |  |  |  |
| Gait exercises |  |  |  |  |  |
| Relaxation exercises |  |  |  |  |  |

1. **Do you think it makes sense to integrate behavioural therapy techniques and coaching approaches into an app for osteoarthritis patients?**

o Yes

o No

o Not specified

1. **Would you consider feedback mechanisms, for example by SMS, to be useful?**

o Yes

o No

o Not specified

1. **Which telemedical possibilities should an osteoarthritis app offer from your perspective?**

- Connection to a teleclinic with 24h possibility of a therapeutic and/or medical consultation
- Connection to the software of the prescribing doctors with the possibility for the patients and/or physiotherapists to contact the physician via chat or video consultation
- Output of collected data and abnormalities on the doctor's PC with own software
- Integration/output of collected data into the hospital IT system
- Output of history data as e-mail attachment
- Output of history data in paper form in the patient file as a hardcopy
- Others, please specify:

__________________________________________________________________

**13.** **For which patient groups do you consider an osteoarthritis app to be useful?**

- Preventive for everybody with BMI>25 and age>65 years
- Available without restrictions for anyone, who want to remain active in their senior years
- For all with osteoarthritis complaints after presentation to the family doctor/ orthopaedic surgeon/ PMR specialist/ pain therapist
- For all osteoarthritis patients, who have received at least 18 therapy units
- For all osteoarthritis patients who have received at least 18 therapy units from an outpatient physiotherapist, after being instructed on how to use the app and how to perform home exercises, which are also the subject of the app
- For all osteoarthritis patients who have already participated in a multimodal therapy programme (e.g. within the framework of in-patient multimodal pain therapy, day clinic physical therapy complex treatment, stay in a rehabilitation centre) for follow-up care and continuous therapy
- Others:

________________________________________________________________________

1. **In your opinion, which digital devices for the patient would improve the offer if they were connected to the app?**

- Fitness bracelets, e.g. for monitoring daily activity
- Digital scale, e.g. for checking weight reduction
- Blood pressure monitors for monitoring blood pressure in the home environment
- Blood glucose meters, for monitoring the blood glucose profile in accompanying diabetes mellitus
- In my opinion, a connection of external end devices does not enrich the therapeutic offer
- A different device would be more helpful (please specify):

_________________________________________________________________________

1. **Would you generally recommend an app to your patients with osteoarthritis?**

- Yes
- No
- Not specified

1. **Please give us the following information about yourself:**

**Gender**

- Male
- Female
- Diverse

**Professional experience**

- Under 5 years
- 5 - 10 years
- 10 - 15 years
- 15 - 20 years
- 20 - 25 years
- 25 - 30 years
- Over 30 years

**What is your occupation?**

- Physician/surgeon

**In which position are you working?**

- Resident/ specialist physician in the inpatient sector
- Resident in the outpatient sector
- Specialist physician in the outpatient sector
- Other professional activity
- Occupational therapist
- Health care and nursing staff
- Health care management
- Masseur
- Medical lifeguard
- Medical assistant
- Medical-laboratory assistant
- Nursing scientist
- Physiotherapist
- Psychologist
- Psychotherapist
- Speech therapist
- Medical student
- Other

**What is your focus of professional activity?**

- Conservative medicine
- Surgical medicine
- Both conservative and surgical medicine
